# Benchmarking Early Unplanned Trauma Re-operations: A Five-Year Institutional Review of 7,400 Orthopaedic Cases

**DOI:** 10.7759/cureus.113074

**Published:** 2026-07-21

**Authors:** Victor Chukwu, Omer Kamal Omer, Abdikarin Elmi, Syeda Zara, Muhammad Rehan Mushtaq, Syeda Mujahid, Anne Baker, Anwar Khan, Georgios Saraglis, Mohamed Arafa

**Affiliations:** 1 Trauma and Orthopaedics, Luton and Dunstable University Hospital, Luton, GBR; 2 Trauma and Orthopaedics, Cambridge University Hospitals, Cambridge, GBR; 3 Trauma and Orthopaedics, Golden Jubilee National Hospital, Clydebank, GBR

**Keywords:** early re-operation, orthopaedic trauma, patient outcomes, patient safety, postoperative complications

## Abstract

Background

Unplanned re-operations within 28 days of trauma surgery play a pivotal role in orthopaedic care and represent a critical indicator of surgical quality and postoperative complications. It has important consequences for patient outcomes and resource allocation in healthcare inefficiencies. This original article investigates over 7,400 orthopaedic trauma cases to detail the incidence, etiology, and timing of early re-operative procedures while presenting contemporary benchmarks for clinical governance and service improvement.

Methods

A retrospective institutional review was conducted of all orthopaedic trauma surgeries performed at Luton and Dunstable Teaching Hospital (LDH) between January 2019 and June 2024. A total of 7,486 procedures were screened, and all unplanned returns to theatre within 28 days (n = 53) were analyzed. Variables collected included patient demographics, American Society of Anesthesiologists (ASA) physical status classification, surgical site, indication, timing, surgeon grade, microbiology, and outcomes. Statistical analysis included exact binomial confidence intervals (CIs), chi-square tests, Welch’s t-tests, and Kruskal-Wallis tests.

Results

Across 7,486 trauma operations, 53 patients required an unplanned return to theatre within 28 days, giving an incidence of 0.71% (95% CI: 0.53%-0.93%). Among re-operated cases, infection was the commonest indication at 40% (95% CI: 26.5%-54.0%), followed by implant/fixation failure at 26% (95% CI: 15.3%-40.3%) and dislocation at 19% (95% CI: 9.4%-32.0%). Age differed significantly by indication for re-operation (Kruskal-Wallis, p = 0.028), with dislocation cases occurring more in older patients. ASA grade also differed by indication (Kruskal-Wallis, p = 0.010). Hip/femur re-operation cases were significantly older than non-hip/femur cases (Welch’s t-test, p < 0.000001). No significant difference in cause distribution by sex was observed (χ², p = 0.486). Most re-operations (72%) occurred between postoperative weeks 2 and 3. Culture positivity was identified in 57% of infection-related cases, predominantly *Staphylococcus aureus* or polymicrobial organisms, with 33.3% of the infection-related re-operated cases resulting in long-term complications or mortality within 24 months.

Conclusion

Early unplanned re-operative procedures are infrequent but clinically significant. The results obtained from this study align with existing UK data and can be used for governance priorities. These findings also highlight priority areas for potential quality improvement, including enhancing infection control measures, improving microbiology turnaround times, optimizing fixation strategies for osteoporotic bone, and improving postoperative surveillance for high-risk populations.

## Introduction

Unplanned early re-operations are universally recognized as a tangible marker of both surgical quality and optimal perioperative care. They highlight complications in the surgical pathway and may represent adverse outcomes that undermine patient recovery, prolong hospital stays, contribute to long-term disability or mortality, and increase healthcare expenditure. Although complications may be unavoidable in some trauma cases due to injury complexity or patient comorbidities, a proportion of early re-operations are potentially preventable through optimization of surgical techniques, infection control pathways, and postoperative surveillance. Evaluating the incidence and contributory factors behind early re-operation is therefore fundamental to improving orthopaedic trauma outcomes and reducing patient morbidity.

Re-operation rates vary widely in the literature, generally ranging between 0.5% and 2% depending on procedure type, patient characteristics, and institutional practices [1-3]. Most early re-operations (defined as within 28-30 days of the index surgery) are attributed to surgical site infection (SSI), mechanical implant failure, or prosthetic dislocation, particularly in patients with hip fracture [4,5]. National registries provide benchmarks against which institutional outcomes can be measured. The UK National Hip Fracture Database (NHFD) reports a re-operation rate of ~1% among hip fracture cases within 30 days, most commonly due to infection and dislocation [6]. There are also reports in the National Joint Registry (NJR) highlighting postoperative infection as the most common driver of early re-operation [7].

While most of the published literature focuses on isolated anatomical regions or specific procedures, such as hip fracture care or lower limb fixation. There remains limited evidence evaluating early re-operations across all orthopaedic trauma procedures within a defined institutional catchment. This study aims to address this important evidence gap by comparing findings from a single-centre study with available national and international literature. Also, while the above-mentioned national registries provide valuable benchmarks for comparison, the importance of single-centre studies cannot be overlooked. These single-centre studies provide significant insights into institutional performance, illustrate differences in local practices, and allow targeted quality improvement initiatives. Local data are also vital to test institutional performance, identify modifiable risks, and promote quality improvement interventions.

This study analyzes five years of early unplanned orthopaedic-related trauma re-operations at Luton and Dunstable Teaching Hospital (LDH), UK, reviewing 7,486 trauma cases done at LDH and comparing findings with available national and international literature. The primary objective of this study was to determine the incidence, causes, and timing of early unplanned re-operations following orthopaedic trauma surgery at our institution over a five-and-a-half-year period. Secondary objectives were to identify patient and surgical factors associated with early re-operation, benchmark institutional outcomes against national and international data, and identify opportunities for intervention, quality improvement, and clinical governance.

## Materials and methods

Study design and setting

A retrospective observational study was conducted at Luton and Dunstable University Hospital, a large district general hospital in the United Kingdom with a busy trauma and orthopaedics department. The study reviewed all trauma operations performed over a five-and-a-half-year period to identify early unplanned re-operations after a primary orthopaedic trauma procedure.

An early re-operation in this study was defined as an unplanned return to theatre within 28 days of the index procedure. The 28-day (four-week) period was selected because it is a widely accepted benchmark for defining early postoperative adverse events and is consistent with quality indicators used by the National Health Service (NHS) and the Royal College of Surgeons for clinical governance, benchmarking, and assessment of surgical outcomes.

This timeframe also aligns with the UK National Hip Fracture Database (NHFD), National Joint Registry (NJR), and NHS Getting It Right First Time (GIRFT) programme, which uses a 30-day window.

Additionally, the 28-day cut-off acts as a threshold to isolate acute postoperative events directly attributable to the index surgery while excluding later complications such as chronic infection and hardware failure arising from longer-term biological or mechanical confounding factors [2].

Study period and population

All orthopaedic trauma surgical procedures undertaken between January 1, 2019, and June 30, 2024, were included. Cases were identified using theatre records and cross-referenced with the departmental trauma database and the hospital electronic patient records (EPR).

Inclusion and exclusion criteria

Inclusion Criteria

Inclusion criteria for this study consisted of adult and paediatric trauma patients undergoing operative management for acute injuries. Only cases where the primary operation was performed at Luton and Dunstable University Hospital were included. Furthermore, all unplanned return to theatre within 28 days of the index procedure was included in the analysis.

Exclusion Criteria

Exclusion criteria for this study included planned staged procedures (such as second-stage surgeries) and elective orthopaedic cases. Patients who returned to the theatre after 28 days were also excluded. Additionally, cases were excluded if the primary operation was performed at an external institution.

Selected Population

Of the 7,486 trauma cases reviewed, 7,433 cases were excluded, while 53 cases met the inclusion criteria.

Data collection

Data were collected using a structured proforma and included the following variables: demographic details (age and gender), American Society of Anesthesiologists (ASA) grade, injury and procedure type, timing of re-operation, indication for re-operation, microbiology results (where applicable), and short- and long-term outcomes.

Data sources included theatre and departmental trauma data systems, clinical notes, operative records, discharge summaries, and microbiology results.

Data analysis

Descriptive statistics were used to evaluate trends and determine the proportion of cases requiring early re-operation. Statistical analysis was performed using proportions with exact (Clopper-Pearson) 95% confidence intervals (CIs). Variables were analyzed using either Welch’s t-test, Kruskal-Wallis tests, chi-square tests, or Fisher’s exact tests, with statistical significance set at p < 0.05.

## Results

Study population and incidence

A total of 7,486 trauma operations were performed at LDH between January 2019 and June 2024. Of these, 53 procedures required an unplanned return to theatre within 28 days of the index surgery, corresponding to a re-operation rate of 0.71% (95% CI: 0.53%-0.93%).

Patient characteristics

**Figure 1:**
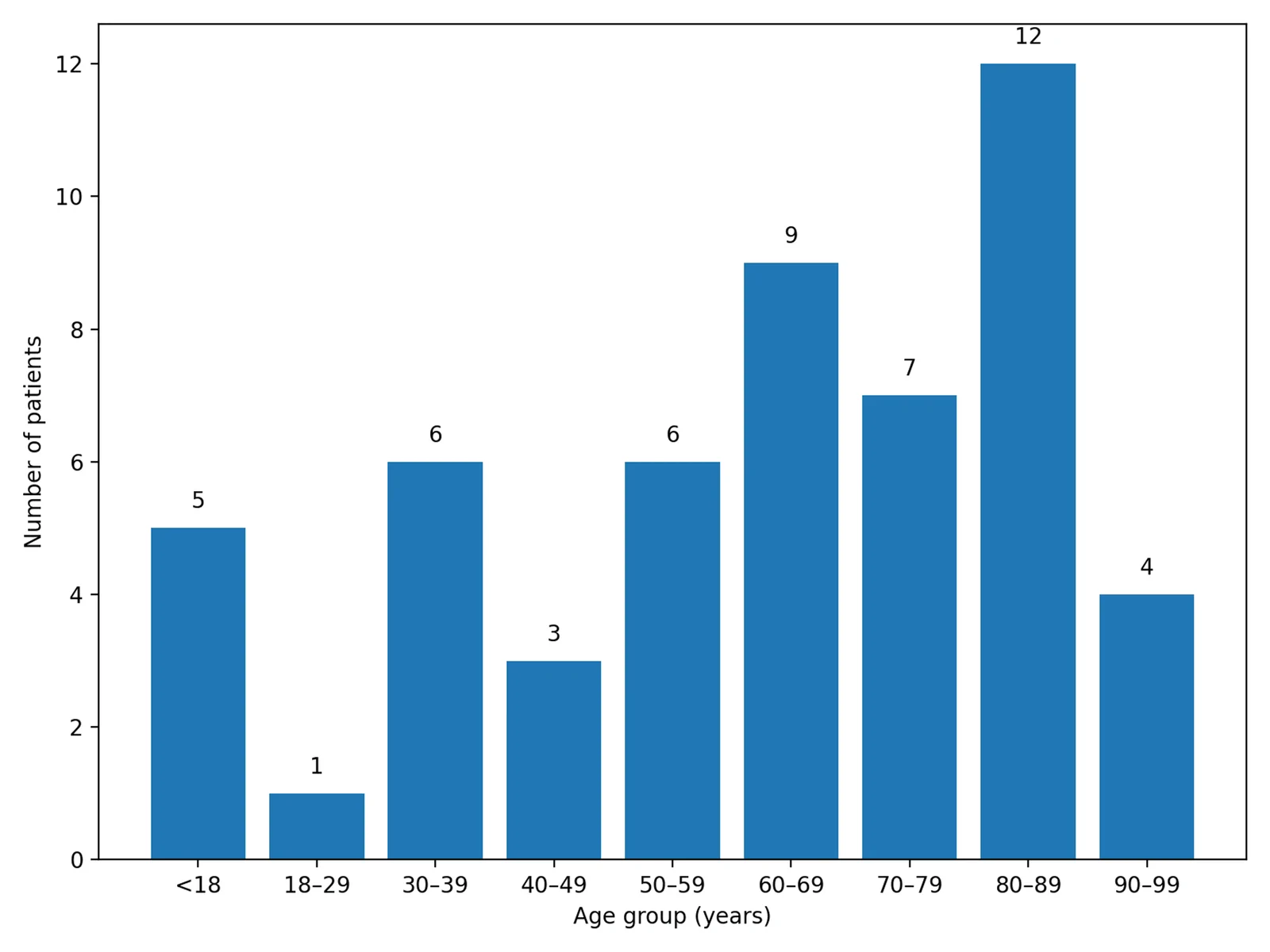
Age distribution of trauma re-operation cases Bars represent the observed proportion of patients in each age category. Sample sizes are shown on the bar of each age category.

**Figure 2:**
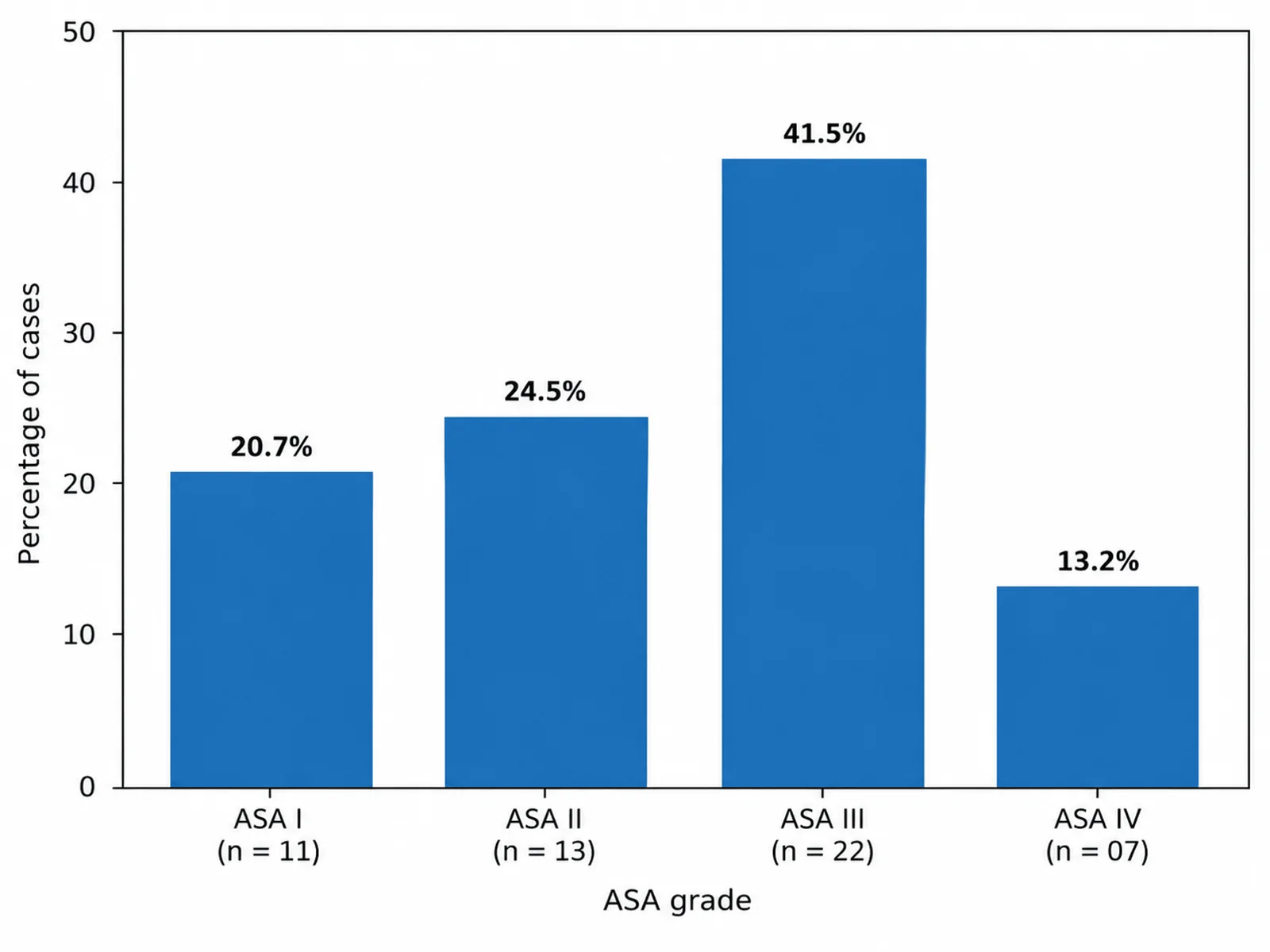
Distribution of ASA grades among patients undergoing early unplanned re-operation Bars represent the observed proportion of patients in each ASA category. Sample sizes are shown beneath each category. ASA: American Society of Anesthesiologists

Patients aged ≥60 years constituted 32 (60%) of the 53 re-operated cases, and 31 (57%) cases were female. As shown in Table 1, Figure 1, and Figure 2, the majority of patients were classified as ASA II-III, suggesting a potential association between moderate anaesthetic risk and the likelihood of re-operation. The mean age of re-operated patients in the study was 62.6 years (standard deviation (SD): 24.6, 95% CI: 55.8-69.4), with a median age of 66 years (interquartile range (IQR): 49-84), indicating that early unplanned return to theatre predominantly occurred in an older patient.

**Table 1:** Patient demographics and clinical characteristics of re-operated patients CI: confidence interval, ASA: American Society of Anesthesiologists

Characteristics (re-operated cases)	Value
Mean age (years)	62.6 ± 24.6 (95% CI: 55.8-69.4)
Age ≥ 60 years	60%
Female	57%
ASA II-III (moderate risk)	66%
Re-operated cases	53
Total cases	7,486
Re-operation rate	0.71% (95% CI: 0.53-0.93)

Indications for early re-operation

**Figure 3:**
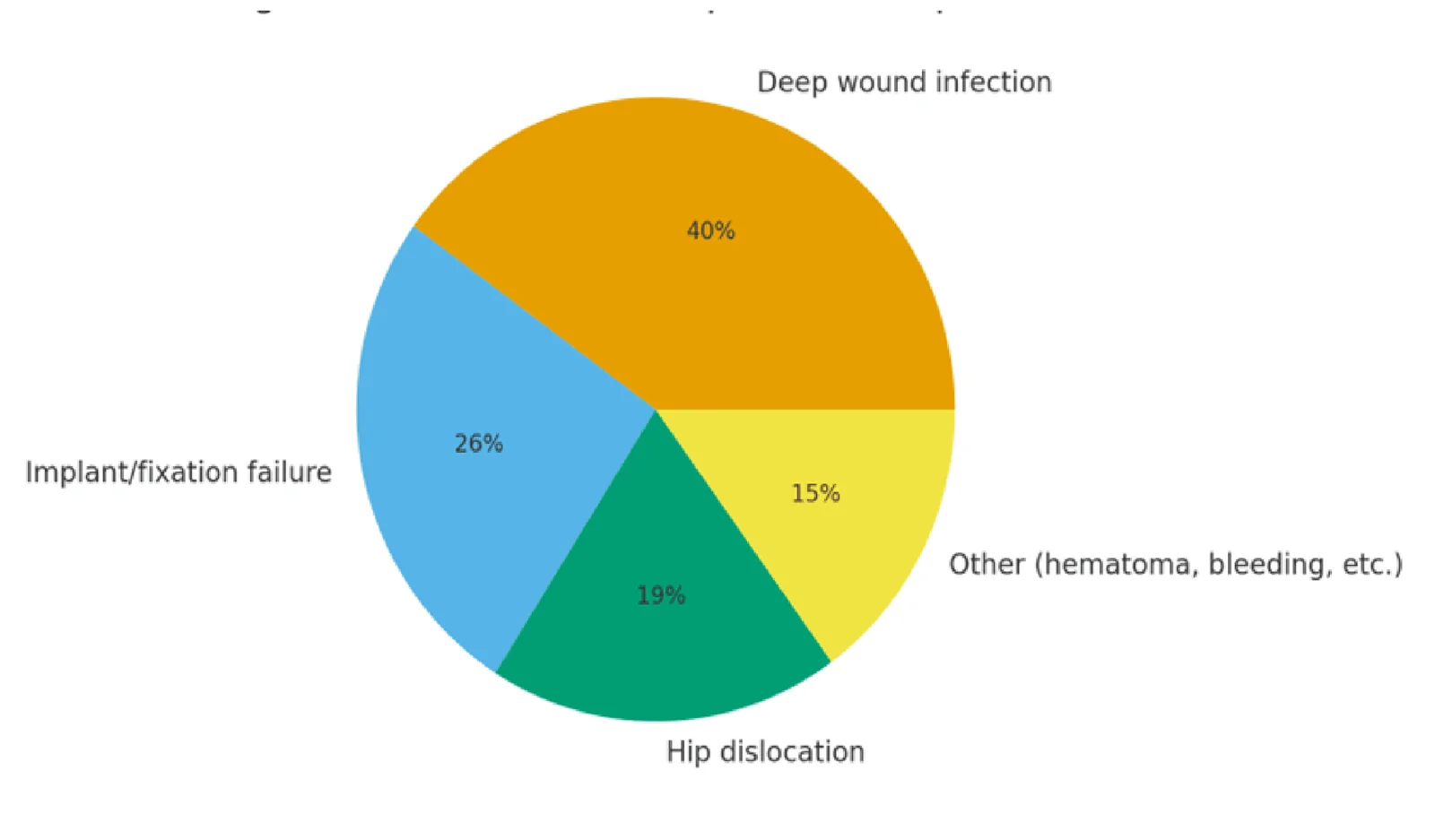
Indications for unplanned early re-operations

The leading causes of unplanned returns to theatre were deep wound infection in 21 (40%) cases (95% CI: 26.5%-54.0%), implant or fixation failure in 14 (26%) cases (95% CI: 15.3%-40.3%), and hip dislocation in 10 (19%) cases (95% CI: 9.4%-32.0%). Less common indications included wound hematoma, compartment syndrome, and postoperative bleeding, which contribute to approximately 15% of early re-operated cases (Figure 3).

**Figure 4:**
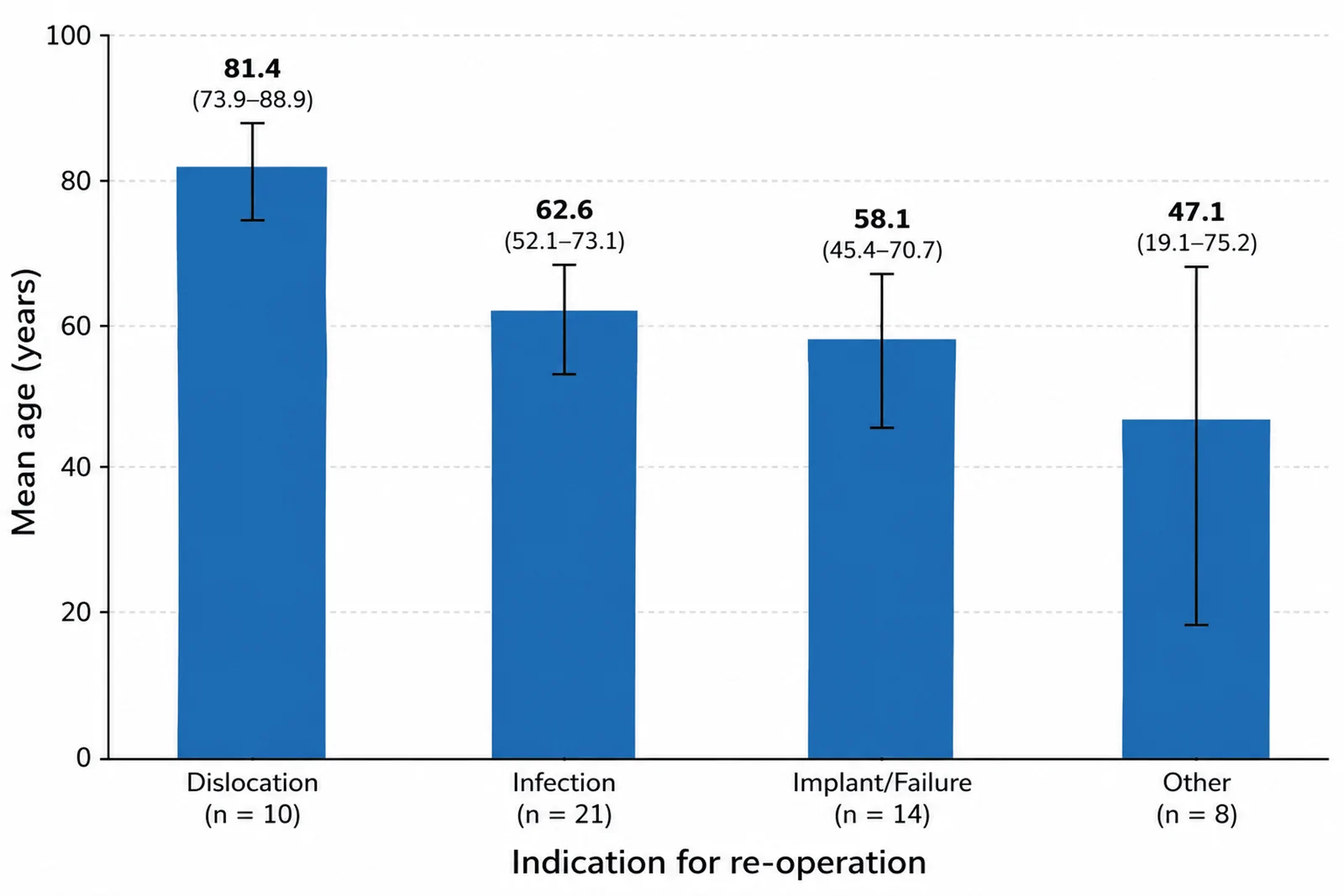
Mean age by indication for early unplanned re-operation Bars represent mean age. Error bars represent two-sided 95% confidence intervals of the mean. Sample sizes are shown beneath each category.

Comparative analysis of age across indication groups demonstrated a significant difference in age distribution (Kruskal-Wallis, H = 9.13, p = 0.028). Mean age was highest in dislocation cases (81.4 years), followed by infection (62.6 years) and implant/fixation failure (58.1 years), while “other” causes occurred in younger patients (47.1 years), indicating that in this study, early unplanned return to theatre due to dislocation was associated with advanced age, whereas miscellaneous causes tend to cluster in a younger patient population (Figure 4).

Considering the recorded ASA values, ASA grade differed significantly by indication for re-operation (Kruskal-Wallis, H = 11.29, p = 0.010). Dislocation and infection-related re-operations were associated with physiologically higher-risk patients, with mean ASA grades of 3.00 and 2.76, respectively, compared to 1.86 for implant/fixation failure and 2.13 for other indications.

Furthermore, using the observed distribution of causes stratified by sex, no statistically significant association was identified between sex and indication for re-operation (χ² = 2.44, p = 0.486), indicating that the pattern of underlying causes did not differ significantly between male and female patients within this cohort.

Anatomical distribution

**Figure 5:**
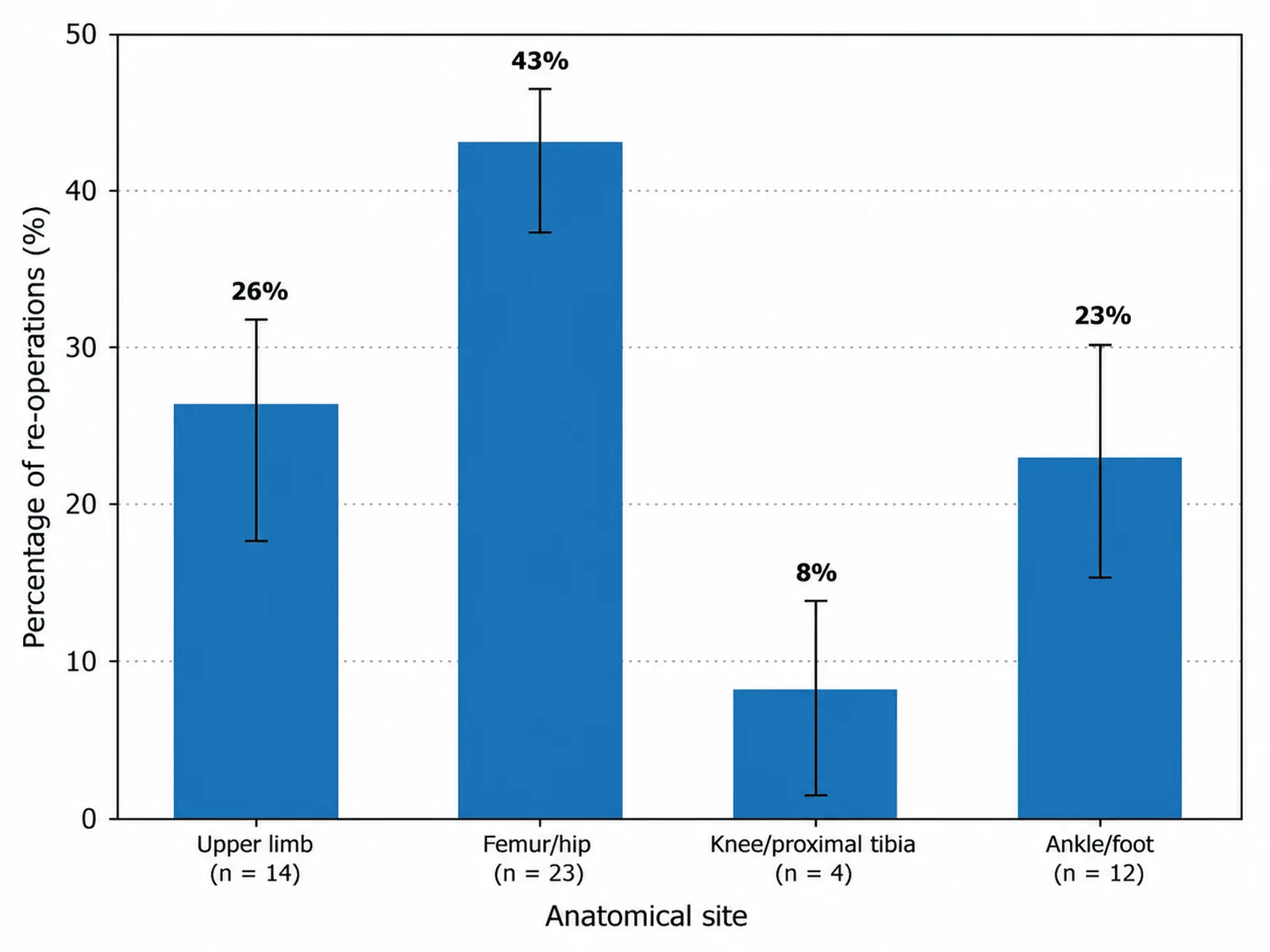
Anatomical sites of cases involved in unplanned re-operations Bars represent the observed proportion of re-operations involving each anatomical site. Error bars represent exact 95% binomial confidence intervals calculated using the Clopper-Pearson method.

Procedures involving the femur and hip accounted for 23 out of the 53 re-operated cases at 43% (95% CI: 28.1%-55.9%) of all re-operations, followed by upper limb procedures (n = 14), ankle/foot procedures (n = 12), and knee/proximal tibia procedures (n = 4). Figure 5 highlights how the anatomical distribution was dominated by hip/femur-related surgeries.

Also, data analysis shows that hip/femur re-operation cases were significantly older than non-hip/femur cases (Welch’s t-test, t = 6.98, p < 0.000001). Re-operations around the hip/femur are concentrated in an older, frailer trauma population with a mean age in hip/femur cases of 81.3 years versus the mean age in non-hip/femur cases of 48.3 years.

Timing of re-operation

**Figure 6:**
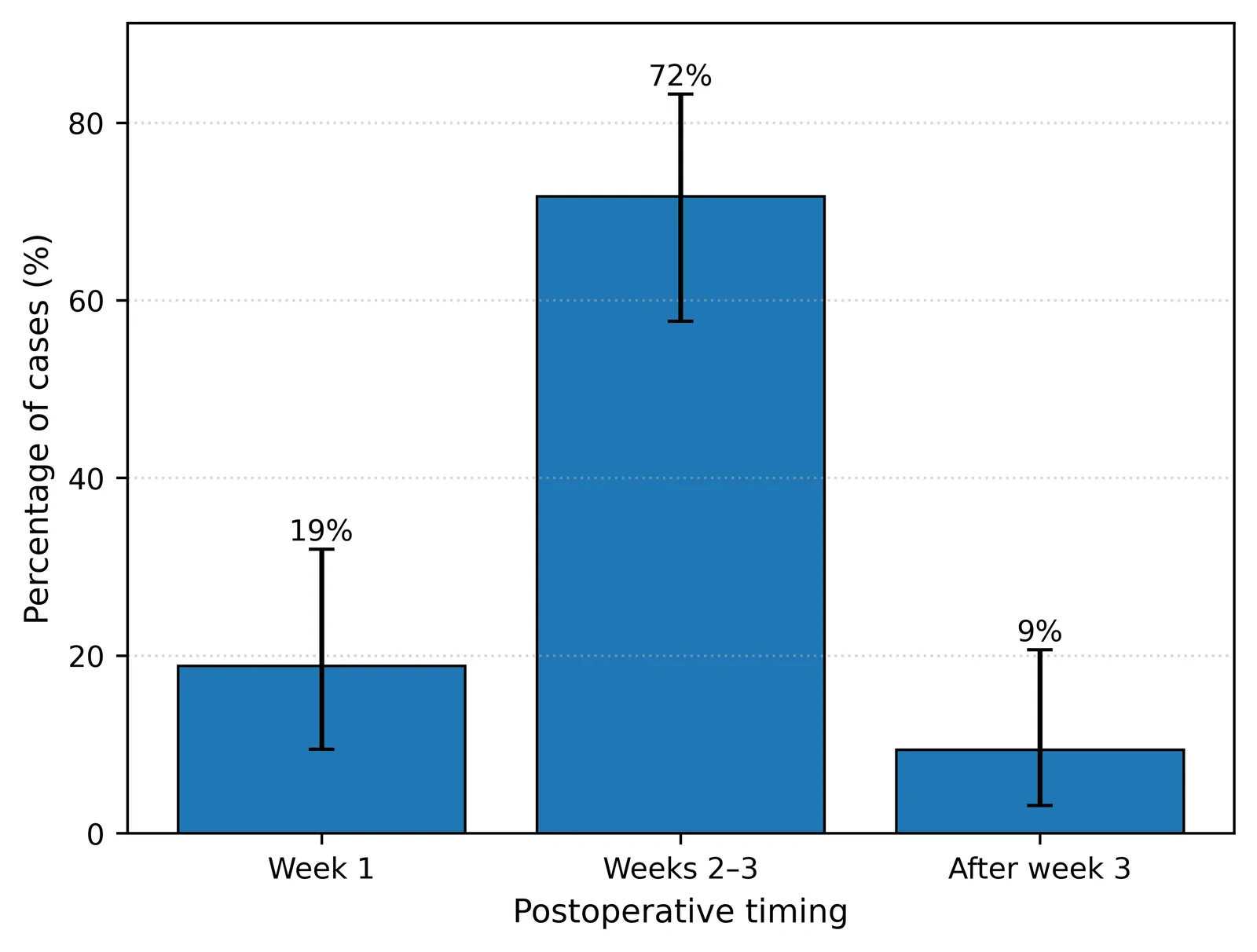
Timing of early unplanned re-operation following the index procedure Bars represent the observed proportion of re-operations occurring within each postoperative interval. Error bars represent exact 95% binomial confidence intervals calculated using the Clopper-Pearson method.

Most unplanned procedures (38 cases) in this study occurred during the second and third postoperative weeks (72%), with a smaller proportion (10 cases) within the first week (19%). Figure 6 shows the incidence of early re-operation clustered during the second and third postoperative weeks.

A summary of all inferential statistical analyses performed in this study is presented in Table 2 and Table 3.

**Table 2:** Comparison of age, ASA grade, and sex by indication for early unplanned re-operation ASA: American Society of Anesthesiologists, IQR: interquartile range, SD: standard deviation, χ²: chi-square test, df: degrees of freedom, M: males, F: females Statistical significance was defined as p < 0.05.

Variable	Infection (n = 21)	Implant/failure (n = 14)	Dislocation (n = 10)	Other (n = 8)	Statistical test	p-value	Result
Age (years)							
Median (IQR)	63.0 (49.0-84.0)	61.0 (44.2-72.8)	82.5 (79.5-87.5)	42.0 (15.0-81.0)	Kruskal-Wallis, H = 9.13	0.028	Significant difference in age across indication groups
Mean ± SD	62.6 ± 23.08	58.1 ± 21.9	81.4 ± 10.4	47.12 ± 33.56	-	-	-
ASA grade							
Median (IQR)	3 (2-3)	1.5 (1-2.75)	3 (3-3)	2 (1.75-2.25)	Kruskal-Wallis, H = 11.29	0.010	Significant difference in ASA across indication groups
Mean ± SD	2.76 ± 0.83	1.86 ± 1.03	3.00 ± 0.67	2.13 ± 0.99	-	-	-
Sex, number (%)							
Female, number (%)	12 (57.1%)	10 (71.4%)	6 (60.0%)	3 (37.5%)	χ² = 2.44, df = 3	0.486	No Significant difference observed in gender across indication groups
Male, number (%)	9 (42.9%)	4 (28.6%)	4 (40.0%)	5 (62.5%)	-	-	-

**Table 3:** Clinical and statistical findings of early unplanned re-operations SD: standard deviation, χ²: chi-square test, df: degrees of freedom Statistical significance was defined as p < 0.05.

Analysis	Category/group	Data	Statistical test	Interpretation
Age comparison by anatomical site	Hip/femur (mean ± SD)	81.3 ± 11.6 years	Welch’s test, t = 6.98, p < 0.000001	Patients undergoing hip/femur re-operation were significantly older than those undergoing non-hip/femur re-operation
	Non-hip/femur (mean ± SD)	48.3 ± 22.3 years		
Timing of re-operation	Week 1	10 (19%)	-	Most re-operations occurred during postoperative weeks 2-3
	Weeks 2-3	38 (72%)		
	After week 3	5 (9%)		
Culture positivity among infection-related re-operations	Positive cultures	12/21 (57.1%)	-	Positive cultures were identified in 57.1% of infection-related re-operations
Long-term complications or mortality among infection-related re-operations	Adverse outcomes	7/21 (33.3%)	-	Approximately one-third of infection-related re-operations resulted in long-term complications or mortality

Microbiological findings

Among infection-related re-operations identified in this study, 12 (57%) cases yielded positive cultures. The predominant pathogens were *Staphylococcus aureus* (including methicillin-resistant *Staphylococcus aureus* (MRSA)), followed by polymicrobial infections.

Outcomes

All cases that underwent re-operation due to infection (n = 21) had a 24-month follow-up assessment performed, using the patient electronic database to evaluate morbidity and mortality. This subgroup was selected because infection was the most common cause of re-operation and is associated with important long-term outcomes, including recurrent infection, further surgery, and prolonged antibiotic treatment. At a mean follow-up of 24 months, 7 (33.3%) of the infection-related cases developed either persistent long-term complications (including chronic infection, non-union, and the need for early prosthetic revision) or mortality.

## Discussion

This study demonstrates that unplanned re-operations within 28 days of trauma surgery at LDH are relatively uncommon, with an incidence of 0.71% (95% CI: 0.53%-0.93%). However, when they occur, they are strongly associated with infection, implant failure, or prosthetic dislocation. These complications disproportionately affect elderly female patients with moderate anaesthetic risk profiles.

Our trauma re-operation rate of 0.71% is broadly in line with UK national benchmarks. The NHFD reports 30-day re-operation rates of ~1% for hip fracture patients [6]. The NJR similarly highlights infection and dislocation as leading causes of early re-intervention following hip arthroplasty [7]. International studies from Europe and North America cite comparable rates of 0.5%-2%, with infection being the most common cause [8-10].

Infection emerged as the most frequent indication for early re-operation in our study, accounting for 40% of cases (95% CI: 26.5%-54.0%). This is consistent with findings from UK and European trauma centres, where infection accounts for 35%-50% of unplanned returns to theatre [11,12]. Our culture-positive rate (57%) is consistent with other notable literature, and the predominance of *Staphylococcus aureus* is well established [13]. These findings reinforce the well-established role of perioperative contamination and highlight the importance of robust infection prevention strategies.

Implant or fixation failure represented one quarter of re-operations in our study and was particularly more common in osteoporotic fractures. This single-centre findings underscore the importance of careful preoperative planning, appropriate implant selection, and consideration of bone quality during fixation, especially in elderly trauma populations.

Hip dislocation in this study represented 19% of all re-operations, with 90% of the identified hip dislocations following a hemiarthroplasty. This aligns with published dislocation rates of 5%-15% following hemiarthroplasty in elderly patients [14,15]. Statistical analysis demonstrated a significant difference in age across indication groups (Kruskal-Wallis, H = 9.13, p = 0.028) in our local study, with dislocation cases occurring in the oldest patients (mean age: 81.4 years). Additionally, ASA grade in this study differed significantly by indication (Kruskal-Wallis, H = 11.29, p = 0.010), with dislocation and infection occurring in more physiologically compromised patients. These findings further support existing evidence linking frailty, comorbidity, and reduced soft tissue stability to increased risk of arthroplasty instability [16].

Importantly, no statistically significant association was identified between sex and indication for re-operation (χ² = 2.44, p = 0.486) in this study, indicating that underlying causes are more likely driven by physiological and mechanical factors rather than gender differences.

The anatomical distribution of re-operations in this study further highlights important clinical patterns. Procedures involving the hip and femur accounted for most unplanned re-operations in our centre at approximately 43% of all cases and were significantly associated with older age (t = 6.98, p < 0.000001).

Timing of re-operation in our study was heavily clustered around weeks 2-3. This supports existing evidence that most surgical site infections (SSIs) and fixation failures manifest in the early postoperative period [17]. The 2-3 week window could also represent a critical period for intensified clinical surveillance to identify cases that could potentially require re-operation and ultimately highlights an important opportunity for timely intervention.

The economic implications of unplanned re-operations are substantial. According to NHS tariff data and the Getting It Right First Time (GIRFT) programme, each orthopaedic re-operation incurs a direct cost often ranging to an estimated value of £7,000-£10,000, excluding indirect costs [14,16]. Infection-related procedures requiring multiple interventions and prolonged antibiotic therapy may exceed £20,000 per patient [14-17]. When indirect costs such as lost productivity, social care, and long-term disability are included, the true economic burden is considerably higher.

Beyond financial considerations, these events carry significant human and service-level consequences, including longer hospital stays, reduced functional outcomes, and reputational effects on institutional performance. Hence, the findings in our study align with national initiatives such as GIRFT, which emphasize reduction of avoidable complications as a key strategy for improving outcomes and reducing healthcare costs.

This retrospective study is not devoid of limitations, particularly its single-centre characteristics, and the 28-day cut-off for evaluation of early trauma unplanned re-operation could potentially overlook late infections, non-union, or fixation failures. As early unplanned re-operation is an uncommon event, restricting the analysis to this clinically relevant time window inevitably resulted in a relatively small number of eligible cases in this study despite screening 7,486 trauma procedures. Furthermore, to ensure that the outcomes reflected the performance of our own institution, we included only patients whose primary trauma procedure was performed at our hospital and excluded patients whose index operation was undertaken elsewhere. This approach allowed us to benchmark institutional performance accurately but may have excluded patients who subsequently underwent re-operation at other hospitals or returned to their primary treating institution. Nonetheless, this study included a large institutional cohort, was conducted over a five-and-a-half-year period, and used a standardized 28-day definition aligned with national benchmarks; the results offer a valuable model for critical benchmarking and clinical review to attain targeted interventions in infection control, operative planning, and postoperative surveillance.

## Conclusions

Early unplanned re-operations following trauma surgery are infrequent but are clinically significant events that provide valuable insight into surgical quality and postoperative complications. In this single-institutional cohort, infection, implant failure, and prosthetic dislocation were the leading causes, with most events occurring within the second and third postoperative weeks.

Despite their low incidence, these unplanned re-operations impose a disproportionate clinical and severe economic burden. Thus, benchmarking local outcomes against national standards, alongside implementing targeted preventive strategies, inevitably serves as a critical approach toward delivering sustained improvements in patients’ postoperative outcomes and healthcare efficiency.
